# Preparation and Structural Evolution of ZrB_2_–HfC–SiC/Dicyanobenzene Hybrid Ultra-High-Temperature Materials Moulded at 250 °C/2 h

**DOI:** 10.3390/ma19132783

**Published:** 2026-07-01

**Authors:** Jiayi Wang, Xiumao Zhu, Xueliang Mu, Bingzhu Wang

**Affiliations:** 1 The Leicester International Institute, Dalian University of Technology, Panjin 124221, China; wjy3142100@mail.dlut.edu.cn (J.W.); 3157284628@mail.dlut.edu.cn (X.Z.); 2 School of Mechanics and Aerospace Engineering, Dalian University of Technology, Dalian 116024, China; bingzhuwang@dlut.edu.cn

**Keywords:** organic–inorganic hybrid, ZrB_2_–HfC–SiC/Dicyanobenzene, Ultra-high-temperature materials (UHMs), oxy-acetylene ablation

## Abstract

**Highlights:**

1. Hafnium carbide and other ceramic powders bonded with phthalonitrile at 250 °C transform into ultra-high-temperature materials capable of withstanding ablation of 0.156 μm/s at 2600 °C 480 s.
2. The evolution patterns of internal temperature and chemical structure of the as-prepared ultra-high-temperature ceramics during ablation are inferred.
3. This work investigates the variations in ablation performance, mechanical properties, and thermal conductivity of the target ultra-high-temperature ceramics during the ablation process.

**Abstract:**

Ultra-high-temperature materials (UHMs) are indispensable for extreme thermal environments (e.g., temperatures exceeding 2000 °C); however, their practical implementation remains severely constrained by demanding processing conditions, including extreme sintering temperatures, prolonged cycles, densification barriers and high equipment cost. In order to meet the low-cost and ablation-resistant requirements of aircraft nose cones, a facile organic–inorganic hybrid strategy is proposed to fabricate ZrB_2_–HfC–SiC composites using a high-char-yield 1,2-dicyanobenzene (DCB) binder, enabling low-temperature moulding at merely 250 °C (2 h; 20 MPa). Upon high-temperature oxidative exposure, the DCB matrix undergoes in situ pyrolysis and synergistic co-sintering with the ceramic powders, producing a multi-layered, self-protective structural architecture. A comprehensive structure–temperature map correlating temperature-dependent phase evolution with flexural strength and thermal conductivity is established, thereby elucidating the underlying self-healing and ablation-resistance mechanisms. The hybrid material in this work exhibits excellent flexural strength, ablation resistance and thermal stability. This study successfully reconciles the long-standing contradiction between low-temperature processability and ultra-high-temperature (2600 °C) service durability, offering a scalable route for next-generation thermal protection systems.

## 1. Introduction

As the Mach number of hypersonic vehicles continues to rise, aerodynamic heating intensifies drastically, posing increasingly severe challenges for thermal protection systems. At critical locations such as the nose cone, leading edges and the combustion chamber and nozzle of scramjets, the material surface temperature can exceed 2000 °C within seconds. This considerably surpasses the maximum service temperature of conventional SiC/SiC and C/SiC composites, precluding their long-term operation in high-temperature, oxygen-rich environments [1,2,3,4,5,6,7,8,9,10]. In recent years, fibre-reinforced ultra-high-temperature materials (UHMs) incorporating Zr-, Hf- and Ta-based transition metal compounds as the matrix have gradually emerged as ideal candidates for thermal protection systems in hypersonic vehicles owing to their excellent high-temperature structural stability, oxidation resistance and ablation resistance [11,12,13,14,15,16,17,18,19,20,21,22].

Processing, however, remains the principal bottleneck in translating material promise into deployable components. High-temperature, pressure-assisted sintering methods, such as hot pressing and spark plasma sintering, typically require temperatures approaching 2000 °C and pressures of 30 MPa, complicating the fabrication of large or geometrically complex components. Polymer-derived ceramic routes demand multiple impregnation–pyrolysis cycles, with ultra-high-temperature ceramic phase formation often requiring temperatures near 2000 °C. Reactive melt infiltration generally operates at similarly extreme temperatures to ensure melt formation and sufficient fluidity. Because all three routes work at or above phase-formation thresholds, decoupling shape formation from high-temperature phase evolution is intrinsically challenging, rendering the reduction of forming temperature a formidable challenge. 

In practical terms, a low-temperature, rapid moulding method compatible with resin-based composite processes would simplify equipment, shorten cycles and reduce cost, thereby enabling scale-up production. Existing organic–inorganic hybrid materials typically employ modified phenolic or polycarbosilane binders to consolidate ceramic fillers and fibres at 200 °C–300 °C for use as coatings, binders and composites; however, their service temperature is often limited to ≤2000 °C. This limitation arises from two principal factors. First, phenolic or polycarbosilane binders provide insufficient char yield, degrading high-temperature strength and ablation resistance [23,24,25,26,27,28,29,30,31,32,33,34,35,36,37,38,39,40]. Second, the hybridisation and ablation-induced structural evolution above 2000 °C remains partially understood, constraining mechanism-guided design for extreme service environments. To date, considerably few researchers in the aerospace field have investigated this area, and almost no publicly available literature exists.

In order to meet the low-cost and ablation-resistant requirements of aircraft nose cones, against the aforementioned backdrop, the present study employs 1,2-dicyanobenzene (DCB) as the binder for ZrB_2_, HfC and chopped SiC fibres. Powder bonding is achieved via hot pressing at 250 °C and 20 MPa for 2 h in air. Moreover, the strategy overcomes (i) the high processing temperatures required for conventional ultra-high-temperature ceramics and (ii) the limited ablation resistance of organic/inorganic hybrid materials.

In this study, X-ray photoelectron spectroscopy (XPS), X-ray diffraction (XRD) and scanning electron microscopy–energy dispersive spectroscopy (SEM–EDS) analyses were performed on the surfaces of ZrB_2_–HfC–SiC/DCB composites subjected to different ablation conditions. Moreover, SEM–EDS analysis was performed on the cross-sections of the composites after ablation at 2600 °C for 480 s. The structural and compositional evolution trends were preliminarily revealed based on these analyses.

## 2. Experimental Section

### 2.1. Materials

DCB (phthalonitrile; formula: 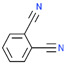; softening point 133 °C–140 °C; purity: 99%) was supplied by the Institute of Chemistry, Chinese Academy of Sciences (Beijing, China).

HfC (D90: 5 µm; purity: 99.5%), ZrB_2_ (D90: 5 µm; purity: 98.5%) and chopped SiC fibres (length: 100 µm; diameter: 10 µm) were obtained from China Yinuo New Material Co., Ltd. (Beidaihe, China).

### 2.2. Preparation of ZHS/DCB Hybrid Materials

**Figure 1:**
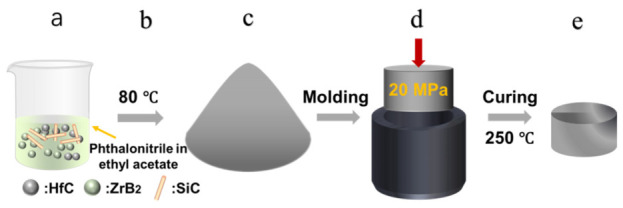
Schematic of the preparation process showing the sequential steps: (a) mixing of raw materials, (b) solvent removal at 80 °C, (c) grinding for homogenisation, (d) hot pressing at 250 °C/20 MPa for 2 h in air and (e) the resulting specimen.

Figure 1 shows the preparation process for the ZrB_2_–HfC–SiC/DCB composite precursor. First, a 20 wt.% phthalonitrile solution in ethyl acetate was blended with ZrB_2_, HfC and chopped SiC fibres in mass ratios of κ:60:60:10 (κ = 25, 50, 75 or 100, corresponding to ZHS/DCB-κ samples (κ = 25, 50, 75 or 100)) (Figure 1). Each precursor mixture was then stirred at 80 °C for 60 min to evaporate the solvent and subsequently ball-milled to achieve a uniform particle dispersion. Grinding was carried out using an F-P400 grinder (Hunan Focucy Experimental Instrument, Changsha, China) at 60 r/min for 20 min. The dried powders were loaded into a graphite die and hot-pressed at 250 °C under 20 MPa for 2 h, producing cylindrical hybrid UHM specimens (Ø ≈ 10 mm; thickness ≈ 12 mm) with high structural homogeneity for subsequent characterisation. ‘Preparation in air or Ar’ refers to moulding the sample in an air or argon environment without applied pressure after removal from the die. 

### 2.3. Characterisation Methods

Crystalline phases were identified via XRD using a PANalytical X’Pert PRO diffractometer (model: 7602 EA, PANalytical, Almelo, The Netherlands) with Cu Kα radiation (λ = 1.5406 Å) operated at 40 kV and 30 mA. Scans were collected over 2θ = 10–80° at a rate of 2° min^−1^. Microstructural features and elemental distributions were examined via SEM (JEOL IT300, JEOL, Tokyo, Japan) coupled with EDS. Room-temperature uniaxial flexural strength was measured in accordance with ASTM C1424 [41] using a universal testing machine, with five specimens per composition to ensure statistical significance. Thermal conductivity at ambient temperature was determined using the transient plane source (TPS) technique (TPS 2500S, Switzerland) according to ISO 22007-2:2022 [42] using a 9 mW heat pulse for 20 s. XPS measurements on the ablated materials were conducted using an AXIS Supra+ instrument (Shimadzu Corporation, Kyoto, Japan).

### 2.4. Ablation Testing 

**Figure 2:**
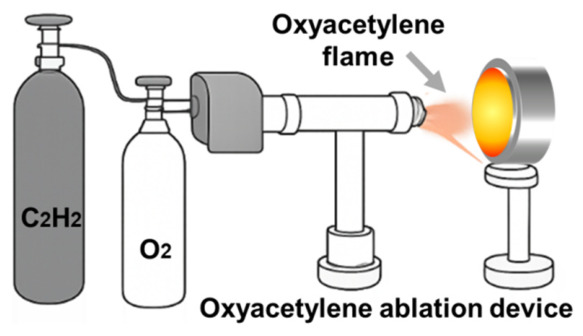
Schematic of the oxy-acetylene ablation experimental setup.

Ablation tests were performed on the organic–inorganic hybrid UHM specimens (Section 2.2) in an oxy-acetylene flame (temperature controlled by the gas flow rates of O_2_ and C_2_H_2_) at 800 °C, 1100 °C, 1400 °C, 1700 °C, 2000 °C, 2300 °C and 2600 °C for 480 s (Figure 2).

Ablation test conditions at 2600 °C were as follows: O_2_ pressure, 0.45 MPa; C_2_H_2_ pressure, 0.12 MPa; gas flow rate, O_2_: 3.37 m^3^/h and C_2_H_2_: 2.36 m^3^/h; nozzle diameter, 3 mm; stand-off distance, 15 mm; heat flux density, 10.1 MW/m^2^; test method, ASTM E285-08(2020) [43].

### 2.5. Porosity Measurement

The porosity was tested via the SEM. The porosity of the pre-test sample was 11.7%. After 60 s of ablation at 2600 °C, it increased to 17.7%, then gradually decreased, changing from 15.3% at 120 s to 13.7% at 240 s, and further to 12.4% at 480 s. 

All tests not marked with a specific standard were performed following the Chinese standards, which specify the sampling size and permissible error. Normally, three to five parallel samples were tested, and the corresponding coefficient of variation was less than 20%.

## 3. Results and Discussion

### 3.1. Effect of Binder Content and UHM Processing Route

To systematically assess the effect of DCB content on the performance of organic–inorganic hybrid UHMs, four formulations were prepared, namely ZHS/DCB-x samples with DCB contents (x) of 25, 50, 75 and 100, respectively. Each batch was evaluated for structural stability and high-temperature ablation resistance to identify the optimal binder ratio.

**Figure 3:**
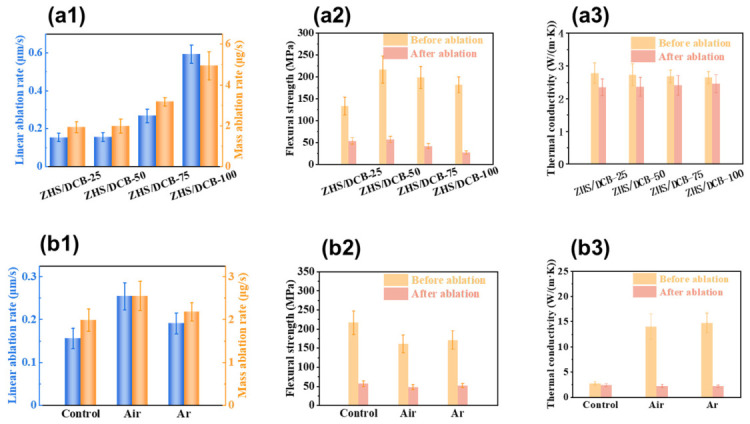
Properties of organic/inorganic hybrid UHMs. (**a**) Effect of 1,2-dicyanobenzene content on properties after ablation at 2600 °C for 480 s: (**a1**) ablation properties, (**a2**) flexural strength and (**a3**) thermal conductivity. (**b**) Effect of different processing routes on properties after ablation at 2600 °C for 480 s: (**b1**) ablation properties, (**b2**) flexural strength and (**b3**) thermal conductivity.

Figure 3 shows that the binder fraction governs ablation, flexural strength and thermal conductivity through the coupled evolution of porosity, viscous glass sealing and ceramic connectivity. At 2600 °C/480 s, the linear recession and mass-loss rates rise monotonically with binder fraction (Figure 3(a1)): 0.153 µm s^−1^ and 1.927 µg s^−1^ for ZHS/DCB-25, increasing to 0.593 µm s^−1^ and 4.944 µg s^−1^, respectively, for ZHS/DCB-100. Higher binder loadings pyrolyse and gasify earlier, generating greater open porosity and larger pores, which elevate the effective oxygen flux and delay the establishment of a continuous, viscous B_2_O_3_–SiO_2_ sealing layer; the net effect is faster material removal.

Flexural strength exhibits a mid-fraction optimum with DCB content (Figure 3(a2)). A moderate binder level (ZHS/DCB-50) promotes sufficient particle necking and contact coordination during forming and early heating, enabling efficient load transfer. At excessively low binder content (ZHS/DCB-25), bonding is inadequate, whereas at excessively high binder content (ZHS/DCB-75 and ZHS/DCB-100), the load shifts to a weaker organic network. After ablation, strength retention is governed by the continuity of the ceramic framework and residual porosity; ZHS/DCB-50 develops a denser, more continuous ceramic–glass layer and therefore retains the highest strength.

Thermal conductivity displays two regimes (Figure 3(a3)). At room temperature and after 2600 °C for 480 s, κ remains essentially unchanged. A higher post-ablation κ principally indicates more continuous heat-transfer pathways and does not necessarily imply improved mechanical robustness.

The processing route further modulates the aforementioned competing effects (Figure 3(b1–b3)). The pressure-controlled route yields the lowest linear recession (0.156 µm s^−1^), the highest room-temperature strength (216 MPa) and the best post-ablation retention (56.5 MPa). Hot pressing under 20 MPa in air produces a denser material, thereby more effectively suppressing oxidation. By contrast, the air/Ar routes without applied pressure tend to generate higher porosity, degrading ablation resistance and strength; their slightly higher κ primarily reflects greater through-thickness continuity of the ceramic network. Combining an intermediate binder fraction (ZHS/DCB-50) with the pressure-controlled route therefore provides the most balanced outcome at 2600 °C for 480 s: low ablation rates, strong retention of mechanical integrity and manageable thermal transport.

### 3.2. Temperature-Dependent Surface Chemistry and Microstructure

Under ultra-high-temperature conditions, the structural evolution of organic–inorganic hybrid UHMs was systematically investigated by establishing a stepwise ablation simulation framework ranging from room temperature to 2600 °C, using the ZHS/DCB-50 formulation as the representative system. By comparing and integrating the results of multidimensional characterisation, the intrinsic coupling between material stability, ablation resistance and microstructure evolution under temperature-dominated conditions was clarified. 

**Figure 4:**
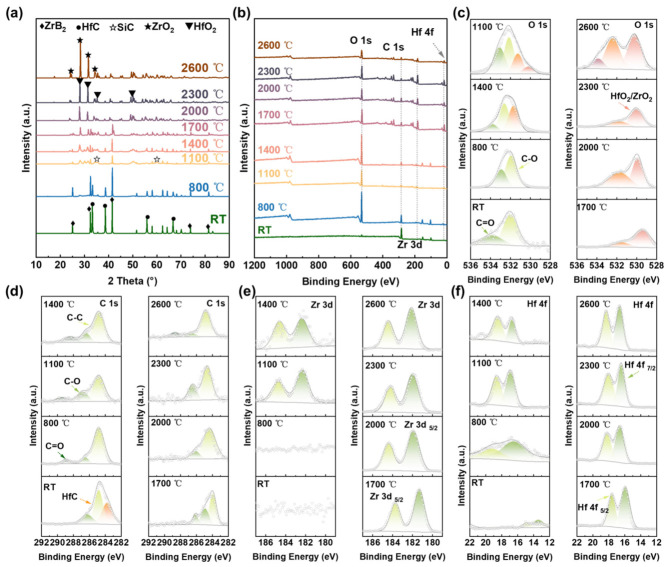
XRD and XPS spectra of ZHS/DCB-50 surfaces after ablation at different temperatures. (**a**) XRD patterns of ZHS/DCB-50 surfaces after ablation at different temperatures. (**b**) XPS survey spectra of ZHS/DCB-50 surfaces after ablation at different temperatures. (**c**) O 1s spectra of ZHS/DCB-50, (**d**) C 1s spectra of ZHS/DCB-50, (**e**) Zr 3d spectra of ZHS/DCB-50 and (**f**) Hf 4f spectra of ZHS/DCB-50.

As illustrated in Figure 4a, the XRD results reveal a distinct temperature-dependent sequence of phase evolution. From room temperature to 800 °C, the diffraction peaks mainly correspond to the initial compositions (HfC (PDF#39-1491), ZrB_2_ (PDF#34-0423) and SiC (PDF#22-1316)), indicating that the material structure remains essentially stable and that oxidation has not yet occurred to any appreciable extent. Between 1100 °C and 1400 °C, diffraction peaks corresponding to HfO_2_ (PDF#34-0104) and ZrO_2_ (PDF#37-1484) begin to appear and coexist with the original peaks, indicating that the oxidation of HfC and ZrB_2_ is partial and the crystalline framework of the material remains continuous. As the temperature increases to 1700 °C, the characteristic SiC peaks weaken markedly, while the oxide peaks intensify, reflecting the parallel thermal decomposition of chopped SiC fibres and continuous oxidation of the primary phases. At 2000 °C, the original carbide/boride peaks are no longer present in the XRD pattern and only ZrO_2_ and HfO_2_ are retained. This progressive disappearance of the initial phases, accompanied by oxidation and melting, indicates that the chemical structure of ZHS/DCB undergoes continuous transformation with rising temperature, resulting in a drastic decline in flexural strength.

To further corroborate the aforementioned phase evolution pathway, the elemental valence states and chemical bonding structure of the sample surface in each temperature region were resolved via XPS analysis (Figure 4b–f). Trace oxygen is detected in ZHS/DCB-50 at room temperature, likely attributable to impurities in the raw materials and moulding at 250 °C. The overall survey spectra show that with increasing temperature, carbide signals diminish and are replaced by C–O, C=O and C–C signals at 800 °C and the C content decreases continuously. Trace O was detected at room temperature, possibly due to the purity of the raw materials and moulding at 250 °C. The O signal strengthens markedly with rising temperature, reflecting the progressive oxidation trend. Owing to coverage by the DCB matrix, no characteristic Zr–B peaks are observed at room temperature; however, upon reaching 1100 °C, Zr–B is gradually oxidised to Zr–O. Furthermore, no Hf–C peaks are detected at room temperature. From 800 °C onwards, Hf–C is progressively oxidised to Hf–O. At 2000 °C and above, carbide and intermediate signals essentially disappear and the spectral features are dominated by metal–oxygen bonding, confirming that the material has transformed into an oxide-dominated ceramic system.

**Figure 5:**
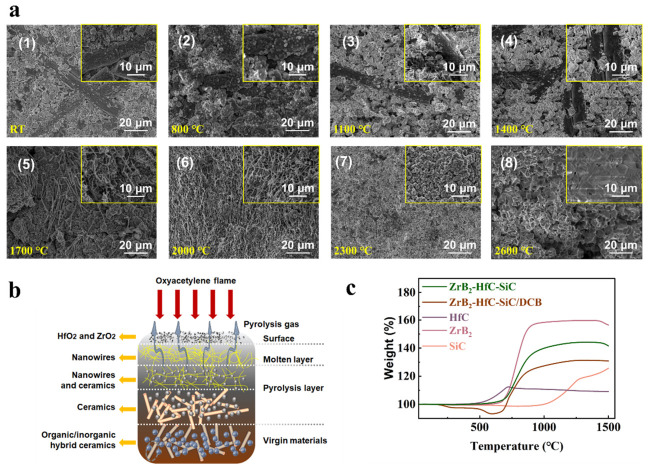
Evolution of ZHS/DCB-50 surfaces after ablation at different temperatures. (**a**) SEM micrographs showing the microstructural evolution of ZHS/DCB-50 sample surfaces after ablation at different temperatures. (**b**) Schematic illustration of the chemical and structural transformation pathways during ablation of hybrid UHMs at different temperature ranges, showing the sequence of decomposition, oxidation and melting processes. (**c**) TG curves of HfC–ZrB_2_–SiC/DCB and its individual components.

Corresponding to the chemical phase evolution, the SEM results (Figure 5a) reveal a hierarchical progression of surface morphology. From room temperature to 800 °C, the surface structure remains dense and uniform. At 1100 °C, porosity increases markedly with cleavage of the organic phase, accompanied by initial inter-particle necking. By 1400 °C, the chopped SiC fibres fracture and decompose, ceramic particles begin to sinter and localised densification develops. At 1700 °C, HfO_2_ and ZrO_2_ adopt a directional arrangement along the gas flow, with ZrO_2_ nanowires forming a continuous fibrous network. By 2000 °C, these nanostructures cover the entire surface and exhibit good alignment and connectivity. Between 2300 °C and 2600 °C, the nanowires partially melt and remodel, ultimately transforming into a continuous oxide glassy overlayer in which pores are further reduced, yielding a structurally intact interfacial layer with excellent thermochemical stability.

Figure 5b shows the schematic illustration of the chemical and structural transformation pathways occurring during the ablation of hybrid UHMs at different temperature ranges, showing the sequence of decomposition, oxidation, and melting processes, consistent with the trend of the porosity test in Section 2.5.

As can be seen in Figure 5c, ZrB_2_–HfC–SiC, ZrB_2_–HfC–SiC/DCB, HfC, ZrB_2_ and SiC exhibit oxidation-induced mass changes. HfC displays a marked mass gain from 497 °C to 728 °C, followed by mass loss, corresponding to oxidation to HfO_2_. ZrB_2_ shows a pronounced mass gain from 595 °C to 931 °C owing to the formation of ZrO_2_ and B_2_O_3_. The subsequent plateau is likely related to molten B_2_O_3_ temporarily suppressing further oxidation. Above approximately 1465 °C, volatilisation of B_2_O_3_ and the degree of oxidation increase and the total mass begins to decrease. SiC exhibits a marked mass gain from 1000 °C owing to the formation of SiO_2_ and CO_2_. The corresponding chemical reactions are as follows.

(1) $$nC_{6}H_{4}{\left(\mathrm{CN}\right)}_{2}\overset{300^{\;{}^{\circ}}C/1h}{\to }{\left[{-C}_{6}H_{4}-(\mathrm{CN})-\right]}_{n}$$

(2) $${\mathrm{HfC}+2O}_{2}\overset{>497^{\;{}^{\circ}}C}{\underset{\mathrm{air}}{\to }}{\mathrm{HfO}}_{2}(s){+\mathrm{CO}}_{2}↑$$

(3) $${\mathrm{ZrB}}_{2}+\frac{5}{2}O_{2}\overset{>595^{\;{}^{\circ}}C}{\underset{\mathrm{air}}{\to }}{\mathrm{ZrO}}_{2}(s){+B}_{2}O_{3}(l)$$

(4) $${\mathrm{SiC}+2O}_{2}\overset{>1001^{\;{}^{\circ}}C}{\underset{\mathrm{air}}{\to }}{\mathrm{SiO}}_{2}(s){+\mathrm{CO}}_{2}↑$$

Based on the insights gained from XRD, XPS and SEM analyses, a three-stage structural evolution mechanism for the ZHS/DCB-50 hybrid UHMs was elucidated. The TG data did not correspond well with the XRD, XPS and SEM results because of the different sensitivity and scale of the process.

### 3.3. Internal Microstructural and Mechanistic Map at 2600 °C

**Figure 6:**
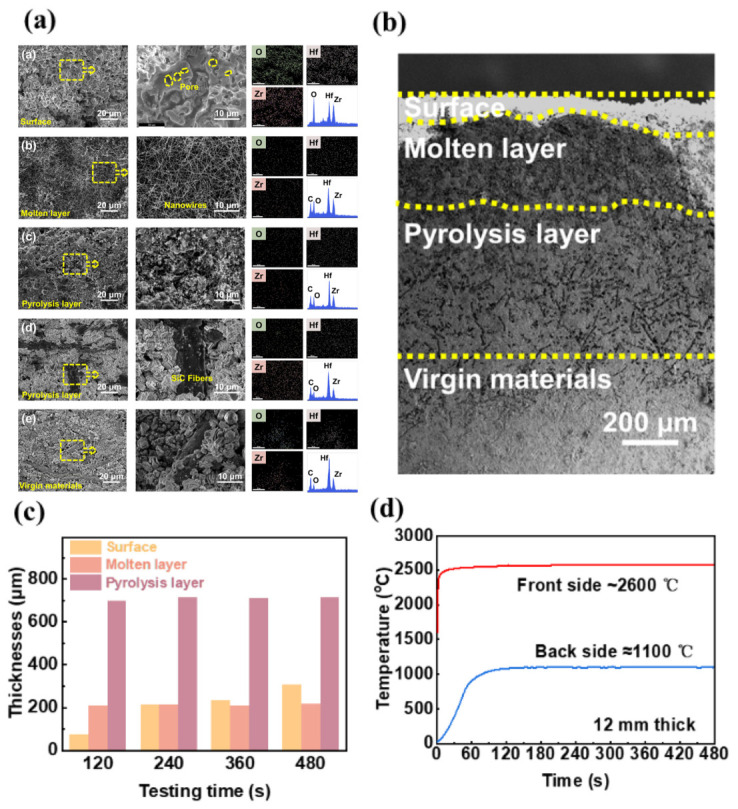
ZHS/DCB-50 after ablation. (**a**) Cross-sectional SEM micrographs of ZHS/DCB-50 at high magnification after ablation at 2600 °C for 480 s. (**b**) Cross-sectional SEM micrographs of ZHS/DCB-50 at low magnification after ablation at 2600 °C for 120 s. (**c**) Thickness evolution of layers during ablation. (**d**) Front and back surface temperatures during ablation.

Figure 6 presents a spatial record of the transient thermal and chemical history during exposure at 2600 °C for 480 s. The cross-sectional stratigraphy in Figure 6a reproduces, in depth, the sequence of surface morphologies observed in the isothermal series presented in Figure 5, thereby establishing a temperature-to-structure map. The compact ZrO_2_/HfO_2_ plus glass-like surface layer corresponds to the 2600 °C state. The next zone is an oriented ZrO_2_/HfO_2_ nanowire forest matching the 2000 °C surface; below this zone, a porous field containing ceramic particles and residual nanowires aligns with the 1700 °C state. Deeper still, partially decomposed chopped SiC fibres and higher porosity mirror the 1400 °C state; and the deepest examined region, with intact chopped SiC fibres, reflects the 1100 °C condition.

As can be seen in Figure 6b,c, the molten oxide crust thickens continuously, reaching approximately 307 µm, which indicates ongoing surface melting, oxidation and re-solidification. In contrast, the thicknesses of the nanowire layer and the transition zone remain essentially constant at approximately 210 and 710 µm, respectively, suggesting the establishment of a quasi-steady-state balance between heat input and material removal. As the pyrolysis gas layer surface recedes, the virgin materials progress into the pyrolysis layer, the pyrolysis layer progresses into the molten layer and the molten layer progresses into the pyrolysis gas layer surface, undergoing the corresponding structural transformations and thereby preserving the overall integrity of the gradient architecture. With prolonged ablation, the ablation layer thickens within the 2600 °C–2100 °C range, whereas the pyrolysis layer thickness remains essentially unchanged in the 2000 °C–1700 °C and 1700 °C–1400 °C ranges. This behaviour arises because the pyrolysis layer is continuously consumed while lower-temperature material is simultaneously replenished from beneath. 

The front- and back-side thermometry in Figure 6d supports the assigned temperature windows. In short, the cross-section represents a frozen profile of moving isotherms, and the thickness of each layer reflects the residence time of each depth interval within its respective temperature window during the test.

### 3.4. Relationship Between Ablation Time and Performance Properties

**Figure 7:**
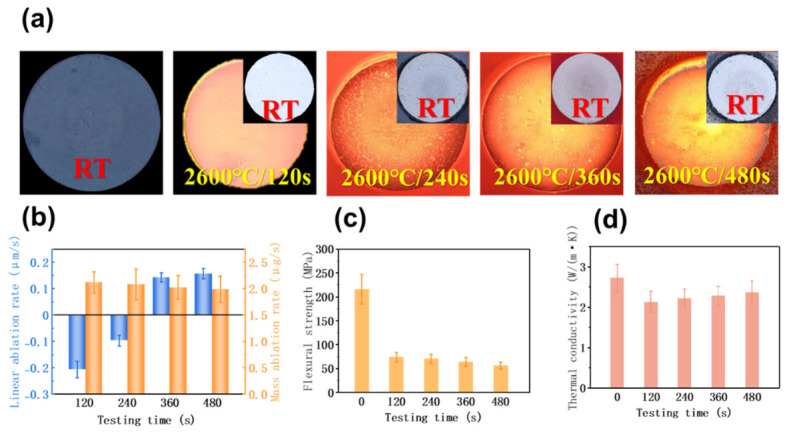
Macroscopic morphology and property evolution of ZHS/DCB-50 during ablation at 2600 °C for different durations. (**a**) Macroscopic morphology evolution, (**b**) ablation property changes, (**c**) flexural strength changes, and (**d**) thermal conductivity changes during ablation at 2600 °C for different durations.

This section systematically examines the time-dependent structural evolution and thermomechanical property changes in the ZHS/DCB-50 formulation after oxy-acetylene flame exposure at 2600 °C for intervals ranging from 0 to 480 s. Macroscopic morphology observations (Figure 7a) showed that the sample surface gradually changed from an initial grey–black colour to grey–white with increasing ablation time, indicating that the surface layer undergoes oxidation at high temperatures and forms a stable oxide cover layer. This colour evolution provides preliminary evidence for the correlation between the material’s appearance and phase transition behaviour, suggesting that the material possesses the capacity to progressively adjust its surface structure upon heat exposure.

Quantitative analysis of ablation metrics (Figure 7b) reveals a distinct two-stage trend in the linear recession rate. During the first 240 s, negative values are observed (−0.207 µm/s at 120 s and −0.097 µm/s at 240 s), reflecting predominantly oxidation-induced volume expansion at the initial stage. Beyond 240 s, linear ablation rates transition to positive values (0.142 µm/s at 360 s, increasing to 0.156 μm/s at 480 s). Meanwhile, the mass ablation rate decreases slightly from 120 s onwards (2.104 → 1.986 μg/s), indicating that the oxide layer gradually densifies and exerts an effective barrier effect under sustained high-temperature exposure, suppressing further material loss.

Mechanical and thermal transport properties also exhibit pronounced time-dependent evolution (Figure 7c,d). Flexural strength rapidly declines from an initial 216 MPa to 73.9 MPa at 120 s, reflecting early degradation of the organic binder and the nascent ceramic framework; subsequently, flexural strength decays more gradually, stabilising at 56.5 MPa by 480 s, which indicates that the oxidised ceramic network retains substantial load-bearing capacity. Thermal conductivity displays a non-monotonic trend: it falls from 2.72 to 2.13 W/(m·K) in the first 120 s as porosity increases and heat-conduction paths are disrupted and then recovers to 2.36 W/(m·K) at 480 s as oxide densification and grain refinement re-establish conduction pathways.

The aforementioned observations collectively define three successive response stages during ablation at 2600 °C: an initial oxidation-induced expansion phase, a transition stage characterised by pore formation and channel penetration and a maturation phase wherein oxide scale growth and structural remodelling stabilise mechanical integrity and thermal transport. The transition from negative to positive linear ablation rates, the gradual reduction in mass-loss kinetics and the non-monotonic recovery of thermal conductivity collectively demonstrate the intrinsic self-regulating and self-protective capabilities of the hybrid UHMs. These findings provide critical theoretical guidance and experimental validation for the optimised design and service-performance enhancement of high-temperature thermal protection materials.

## 4. Conclusions

In this study, a robust organic–inorganic hybrid strategy was employed for the low-temperature fabrication of ZrB_2_–HfC–SiC composites using a phthalonitrile (DCB) binder, consolidated at only 250 °C. The optimised ZHS/DCB-50 composite demonstrates a promising room-temperature flexural strength of 216 MPa and remarkable thermal stability, evidenced by a low linear recession rate (0.156 µm s^−1^) and mass-loss rate (1.986 µg s^−1^) after 480 s of ablation at 2600 °C. Multiscale characterisation (XRD, XPS and SEM) elucidates a three-stage structural evolution: (i) T < 1100 °C, characterised by polymer pyrolysis and the formation of an embryonic ceramic skeleton, (ii) 1100–1700 °C, governed by initial oxidation and viscous sintering of the ceramic phase, and (iii) T > 1700 °C, dominated by the in situ growth of ZrO_2_/HfO_2_ nanowire arrays accompanied by local vitrification. This coupled oxidation–sealing process forms a multi-layered, self-protective scale that effectively reconciles low-temperature moulding with extreme-temperature serviceability. The established structure–temperature map provides a quantitative mechanistic framework, offering critical guidance for the design and deployment of high-performance thermal protection systems in hypersonic environments.

## 5. Patents

This research has obtained a Chinese-granted patent (Patent No. ZL202511323736.7).

## Data Availability

The original contributions presented in this study are included in the article. Further inquiries can be directed to the corresponding author.
